# Assessing the Use of PCR To Screen for Shedding of Salmonella enterica in Infected Humans

**DOI:** 10.1128/JCM.00217-20

**Published:** 2020-06-24

**Authors:** Tyler Lloyd, Monica Bender, Sandra Huang, Robert Brown, Rita Shiau, Emily Yette, Munira Shemsu, Mark Pandori

**Affiliations:** a Alameda County Department of Public Health, Division of Communicable Disease Control and Prevention, Public Health Laboratory, Oakland, California, USA; b Alameda County Department of Public Health, Division of Communicable Disease Control and Prevention, Acute Communicable Disease Control, Oakland, California, USA; c Nevada State Public Health Laboratory, University of Nevada, Reno, School of Medicine, Reno, Nevada, USA; Brigham and Women's Hospital

**Keywords:** clearance, PCR, public health, *Salmonella*, stool, viability

## Abstract

Recovery from enteric bacterial illness often includes a phase of organismal shedding over a period of days to months. The monitoring of this process through laboratory testing forms the foundation of public health action to prevent further transmission. Regulations in most jurisdictions in the United States exclude individuals who continue to shed certain organisms from sensitive occupations and situations, such as food handling, providing direct patient care, or attending day care. The burden that this creates for recovering patients and their families/coworkers is great, so any effort to provide efficiency to the testing process would be of significant benefit. We sought to assess the ability of PCR for the detection of Salmonella enterica shedding and to compare that ability to culture-based testing. PCR would be faster than culture and would allow results to be generated more quickly. Herein, we show data that indicate that, while PCR and culture testing agree in the majority of cases, there are incidents of discordance between the two tests, whereupon PCR shows positive results when culture indicates lack of detectable viable organisms. Using culture-based testing as the standard, the negative predictive value of PCR was found to be 100%, while the positive predictive value was 79%. The nature of this discordance is briefly investigated. We found that it is possible that PCR may not only detect nonviable organisms in stool but also viable organisms that remain undetectable by standard culture methods.

## Abstract (precis)

Recovery from enteric bacterial illness often includes a phase of organismal shedding over a period of days to months. The monitoring of this process through laboratory testing forms the foundation of public health action to prevent further transmission. Regulations in most jurisdictions in the United States exclude individuals who continue to shed certain organisms from sensitive occupations and situations, such as food handling, providing direct patient care, or attending day care. The burden that this creates for recovering patients and their families/coworkers is great, so any effort to provide efficiency to the testing process would be of significant benefit.

## INTRODUCTION

While bacteriologic culture remains the mainstay for the detection and identification of enteric pathogens, the technological landscape is changing. The advent and use of nucleic acid amplification tests (NAAT) for the detection of such pathogens in human specimens may provide more sensitivity and speed than culture for this purpose. It is notable that culture-independent diagnostic testing (CIDT) will not result in the establishment of an isolate. While this may not impact the medical management of an infected individual, the failure to generate a bacterial isolate prevents public health laboratory efforts to further describe certain organisms. Descriptors such as serotype and drug susceptibility are more difficult and, in some cases, impossible to discern from noncultured specimens alone. Among the diseases most impacted by CIDT is infection with *Salmonella* bacteria.

Salmonella enterica infection remains common in the United States despite over a century of public health effort (1–3). Among the tools used to combat the spread of this agent is the exclusion of infected individuals in sensitive occupations or situations, such as food handling, direct patient care, or day care attendance (4). Many state and local public health jurisdictions in the United States require by regulation that persons with *Salmonella* infections be excluded from their sensitive occupation or situation until multiple stool samples are tested and found to be free of S. enterica. In Alameda County, patients are cleared to return to their sensitive occupation or situation when two consecutive stool specimens collected at least 24 h apart test negative for *Salmonella* by culture conducted at the local public health laboratory. For patients who are infected with *Salmonella* and awaiting clearance of shedding are being held out of work, child care, or day care until cleared, it is highly desirable to ascertain clearance/lack of infectivity as quickly as possible. CIDT may offer such an advantage over culture, as specimens can be prepared and tested by way of nucleic acid amplification more quickly than culture-based testing.

We sought to compare the use of PCR to culture-based testing for the detection of shedding of S. enterica in patients previously diagnosed. Multiple specimens from these patients were provided in chronological sequence in furtherance of clearance for return to work or day care. Specimens were evaluated by both PCR and standard culture techniques in furtherance of comparing the two methods for assessing for the presence of a *Salmonella* organism. Our second objective was to investigate the following two hypotheses for discordance found between PCR and culture methods: (i) PCR detects the genomes of nonviable organisms; and/or (ii) PCR is more sensitive at detecting *Salmonella* cells regardless of viability. We sought to evaluate the latter possibility through the use of spiked stool samples.

## MATERIALS AND METHODS

Culture for *Salmonella* was performed from stool samples received in ParaPak culture and sensitivity (C&S) ParaPak Enteric Plus transport media (Merdian Biosciences). Selenite broth (Remel) (9 ml) was inoculated per manufacturer instructions and incubated for18 to 24 h. Selenite broth was subcultured to XLD (xylose, lysine, desoxycholate; Remel) and HardyChrom *Salmonella* plates (Hardy Diagnostics). Subcultures were incubated in ambient air at 35°C for 18 to 24 h and then examined for colonial morphology typical of S. enterica. Colonies resembling *Salmonella* were confirmed with *Salmonella* polyvalent O antisera (Remel).

The following criteria for specimen acceptability that was utilized are per instructions for the Cary-Blair ParaPak C&S ParaPak Enteric Plus transport media. Patients are instructed to transfer their stool to the sample container containing preservative to form a slurry up to a “fill here” line on a modified Cary-Blair specimen tube. Specimens must be subcultured within 96 h of that collection. Specimens that do not meet these criteria are noted, and any negative stool samples that do not meet collection criteria are labeled with a disclaimer indicating that a negative result may be unreliable. No specimens used in this study are known to have been collected or handled outside these criteria.

Detection of *Salmonella* by PCR was performed from an aliquot of the same inoculated selenite broth used for culture. Nucleic acid was extracted and purified using a Roche MagNAPure compact system (170 μl of broth was used for the extraction with 210 μl of Roche lysis buffer and 20 μl of Roche proteinase K solution; nucleic acid was eluted at a volume of 100 μl). Extracted *Salmonella* DNA was amplified/detected using real-time PCR on an ABI 7500 instrument (Thermo Fisher, Waltham, MA) with a kit of analyte-specific reagents for detection of S. enterica (Liferiver). The amount of time spent performing PCR was monitored manually and was found routinely to require between 5 and 6 h to accomplish identification/detection (after overnight selenite incubation). Culture-based results fell into the following three categories with regard to time for definitive detection and identification (after overnight selenite incubation): those that required 40 to 48 h after selenite incubation, those that required 62 to 72 h after overnight selenite incubation, and those that took from 84 to 120 h to complete identification.

Experiments on the sensitivity of PCR versus culture to detect viable *Salmonella* used selenite broth inoculated with stools that contained no *Salmonella*. Stools in selenite broth were inoculated with S. enterica serovar *typhimurium* (S. typhimurium) ATCC 14028, serially diluted with stool-selenite mixture at 1.5 × 10^8^ to 1.5 × 10^−2^ CFU. Samples were tested identically to the *Salmonella* testing protocol above. Selenite broths which yielded PCR-positive but culture-negative results were placed back into the incubator overnight, plated, and observed for *Salmonella*. If the plates were again negative, the original selenite was incubated overnight, plated, and observed again for a total incubation time of 72 h. Plated culture results recorded at 24, 48, and 72 h of incubation are based on the total time incubated of the original selenite broth.

## RESULTS

Sixty-four Salmonella clearance specimens collected chronologically from 13 patients were tested by both culture and PCR (Table 1). Overall, the agreement between PCR and culture was 86% (55/64 specimens). Utilizing culture-based testing as a standard, the negative predictive value (NPV) for PCR was 100%. The positive predictive value (PPV) was 79%. Discordant specimens (those found reactive by PCR but culture negative) were calculated to possess a mean real-time PCR cycle threshold (*C_T_*) value of 30.3 with a range of 23.8 to 34.6. Concordant specimens had *C_T_* values in the range of 17.0 to 33.4. In five of nine discordant instances, discordant specimens were followed by a specimen that showed concordance and would not have delayed clearance (e.g., case F). In 2 of 13 patients (15%; cases C and L) PCR results were discordant with culture such that a culture would have indicated resolution of infection, while the PCR would have indicated continued shedding. In 11 of 13 cases (85%), no differences in clearance process outcomes would have been caused through the use of PCR in lieu of culture.

**TABLE 1:** Characteristics of *Salmonella* cases that underwent PCR and 24-h culture

Case	No.	Day stool collected	Culture result	PCR result	Sal^*a*^ *C_T_*	IC^*b*^ *C_T_*
A	1	0	−	+	32.8	32.1
	2	2	+	+	21.2	Undet^*c*^
	3	18	−	−	Undet	30.7
	4	19	−	−	Undet	32
B	1	0	+	+	30.9	32.2
	2	24	−	−	Undet	31.3
	3	26	−	−	Undet	31.8
C	1	0	+	+	21	Undet
	2	2	+	+	25	32.3
	3	7	+	+	26.6	31.1
	4	9	+	+	25.9	31.6
	5	15	+	+	27.1	32.4
	6	16	+	+	25	33.8
	7	21	−	+	28.2	30.5
	8	23	−	+	28.1	30.2
D	1	0	+	+	33.4	17.6
	2	1	+	+	19.1	31.1
	3	5	+	+	18.4	33
	4	6	+	+	21.8	11.5
	5	14	−	−	Undet	15.9
	6	15	−	−	Undet	17
E	1	0	+	+	22.6	31.7
	2	0	+	+	22.4	32.4
	3	5	+	+	21.9	31.2
	4	6	+	+	22.2	31.8
	5	26	+	+	23.9	18.5
	6	27	+	+	22.1	14
	7	33	+	+	29.2	18.6
	8	35	+	+	20.9	18.1
	9	40	+	+	18.9	32.4
	10	42	+	+	20.7	18.9
	11	48	+	+	23	15.4
F	1	0	+	+	24.1	32.6
	2	1	+	+	21	33
	3	8	+	+	27	31.7
	4	9	+	+	19.2	Undet
	5	15	−	+	29.6	30.5
	6	16	+	+	23.3	34
	7	19	−	+	28.8	32.4
	8	20	+	+	26.3	32.2
	9	28	−	−	Undet	32.1
	10	29	−	−	Undet	31.8
G	1	0	−	−	Undet	17.7
	2	0	−	+	23.5	10.7
	3	1	−	−	Undet	16.5
H	1	0	−	−	Undet	19.4
	2	1	−	−	Undet	18.3
	3	9	+	+	23.4	22.1
I	1	0	+	+	17	32.3
	2	1	+	+	20.8	18.2
	3	8	+	+	18.1	32.2
	4	9	+	+	17.6	33.1
J	1	0	−	+	33.5	17.6
	2	1	−	−	Undet	22.9
	3	6	−	−	Undet	13.7
K	1	0	−	−	Undet	31.8
	2	0	−	−	Undet	31.9
	3	7	−	−	Undet	26.2
L^*d*^	1	0	−	−	Undet	16.7
	2	1	−	+	27.1	18.1
	3	2	−	+	31.2	15.7
M	1	0	−	−	Undet	16.5
	2	1	−	−	Undet	12.2
	3	17	−	−	Undet	19.5

a Sal, *Salmonella* PCR.

b IC, internal PCR control.

c Undet, undetectable.

d This patient was treated with antibiotics.

To better understand the discordance between culture and PCR, we evaluated three stool samples in selenite broth, spiked with 10-fold serial dilutions of S. enterica grown in liquid culture. All spiked specimens were subjected to culture and PCR as above. Results for all stools at all dilutions are shown in Table 2. For stool 1, discordance between PCR and 24-h culture can be seen at the four highest dilutions tested. For the five highest dilutions in that stool, we allowed the stool to incubate in the selenite broth for 48 h, and for the two highest dilutions (0.15 and 0.015 CFU/ml), we allowed the stools to incubate in selenite broth for 72 h. For the 15-CFU/ml and 1.5-CFU/ml dilutions, only the 48-h method of culture revealed the presence of viable *Salmonella*. The 0.15-CFU/ml dilution was positive by culture for *Salmonella* only when using the 72 h of incubation of stool with selenite broth. Two additional stool specimens from different sources were utilized to replicate the experiment. Unlike stool 1, repetitions of the experiment (stool 2 and stool 3) (Table 2) demonstrated concordance between culture and PCR using the standard 24-h culture methods.

**TABLE 2:** Test results of spiked stool specimens

Stool no. and amt (CFU/ml)	Result of test:			
	24-h culture^*a*^	PCR	48-h culture	72-h culture
Stool 1				
1.5 × 10^7^	+	+	NA	NA
1.5 × 10^6^	+	+	NA	NA
1.5 × 10^5^	+	+	NA	NA
1.5 × 10^4^	+	+	NA	NA
1.5 × 10^3^	+	+	NA	NA
1.5 × 10^2^	+	+	+	NA
15	−	+	+	NA
1.5	−	+	+	NA
0.15	−	+	−	+
0.015	−	+	−	−
Stool 2				
1.5 × 10^7^	+		NA	NA
1.5 × 10^6^	+		NA	NA
1.5 × 10^5^	+		NA	NA
1.5 × 10^4^	+		NA	NA
1.5 × 10^3^	+		NA	NA
1.5 × 10^2^	+		NA	NA
15	+	+	NA	NA
1.5	+	+	NA	NA
0.15	+	+	NA	NA
0.015	−	−	+	NA
Stool 3				
1.5 × 10^7^	+		NA	NA
1.5 × 10^6^	+		NA	NA
1.5 × 10^5^	+		NA	NA
1.5 × 10^4^	+		NA	NA
1.5 × 10^3^	+		NA	NA
1.5 × 10^2^	+		NA	NA
15	+	+	NA	NA
1.5	+	+	NA	NA
0.15	−	−	−	−
0.015	−	−	−	−

a +, positive by culture or reactive by PCR; −, negative; NA, not applicable.

## DISCUSSION

The data shown herein indicate that PCR and culture-based testing are not necessarily equivalent or interchangeable for the testing of *Salmonella*-infected patients in the course of illness resolution. For specimens tested in chronological series from infected patients, several instances of PCR reactivity were observed in specimens which were negative by standard 24-h culture methods. Such discordance perhaps should not be a surprising finding based on observations made in other bacterial infections. The detection of Mycobacterium tuberculosis genomes by PCR continues for long after the ability to detect viable M. tuberculosis organism has ceased during treatment (5). Similarly, treatment of chlamydia or gonorrhea results in the shedding of nonviable organismal components for several days (6, 7). PCR and culture are inherently different tests which evaluate different aspects of organisms. One detects specific DNA molecules, which can be biochemically stable in the absence of organismal viability, while culture-based testing depends on the growth of a living organism. An interesting component of the medical management of *Salmonella* that puts it in contrast to tuberculosis and sexually transmitted disease (STD) management is that *Salmonella* is not routinely treated by antibiotics in uncomplicated/low-risk patients (4). Thus, any discordance caused by viability would be considered to be generated by agent or host factors and not therapy. Of the cases included herein, only one of them involved antibiotic treatment (case L) (Table 1). For that reason, the discordance observed in this study may not have been caused by lack of viability due to antimicrobials; lack of viability caused by host immune factors remains a possibility. Another possible mechanism of discordance between viability (culture) and PCR might be caused by the time between collection and analysis. It is notable that all human specimens analyzed in this study were received by the laboratory at less than or equal to 48 h post-specimen generation. The two exceptions were received at 72 h (case A, specimen 1, and case C, specimen 7). Both specimens demonstrated discordance between culture and PCR.

In the realm of public health policy, it is usual practice that individuals who do not possess detectable, culturable organism can be cleared to return to public interaction. The data included herein indicate that it may be possible that such people are still shedding viable organism that is simply not detectable by standard 24-h culture methods. Whether the shedding of such low numbers of organism is relevant in the transmission of salmonellosis is unknown for humans. The infectious dose of *Salmonella* is largely considered high, approximately 100,000 CFU or higher, implying that patients found negative by culture would be significantly less able to transmit infection (8). The data in this study revealed that the nature of the discordance between culture and PCR is uniform; PCR was never observed to be negative in the case of a positive standard 24-h culture. Noting this, it is perhaps possible that PCR could play a role in the clearing of cases by use of a testing algorithm. If specimens submitted for clearance were first screened by PCR, a negative result could be confidently discerned as a true-negative specimen lacking the presence of viable *Salmonella* cells. A positive PCR could reflex, however, to a culture test to ascertain for the presence/absence of viable *Salmonella* cells. In this way, patients who are truly negative could be cleared 1 to 4 days earlier using PCR, while positive cases would not be inappropriately cleared. For public health jurisdictions that require testing of close human contacts to patients, this would be a particularly useful strategy, as the vast majority of contacts screen negative for *Salmonella* (>95% in Alameda County over 2016 to 2018).

This study reveals that the *C_T_* value of specimens subject to PCR may provide some intelligence regarding individual clearance cases that culture does not. The *C_T_* value is the measure of the cycle-moment in PCR that a specimen goes from negative to positive, and that value is inversely proportional to the amount of DNA target being detected. For clearance/shedding specimens collected in chronological series, a progressive increase in *C_T_* values might indicate a process toward ultimate clearance, while continuously unchanged *C_T_* values might indicate an absence or some difficulty in physiological clearance. Such information may be of value to public health investigators.

While studies have been performed in animals, there is a notable lack of peer-reviewed, published work on the topic of *Salmonella* clearance in humans (9–12). Therefore, it is not clear how long people infected with *Salmonella* spp. typically shed organism during the course of their illness or how antibiotics might affect this, although at least one study has sought to evaluate this (10, 13). It is also unknown whether the data reflected in the cases in this work are reflective of the norm. Moreover, it is not known whether there is a genetic differential in the resolution of infection among *Salmonella* serotypes, with some serotypes being more difficult to resolve than others (9). Additionally, it could be hypothesized that this study may have been confounded if any of the cases evaluated were on antibiotic therapy for any reason and that this was hidden from the authors of this work. As indicated, all but one of the cases studied herein were deemed as uncomplicated and were not treated with antibiotics. Pharmacological treatment in a manner that would be unknown to the investigators may have affected the viability of organisms in feces and would have been a possible cause of discordance with PCR. This study has started to elucidate the importance of *Salmonella* clearance in a modern public health setting where CIDTs are becoming commonplace.
